# Characteristics of daily life gait in fall and non fall-prone stroke survivors and controls

**DOI:** 10.1186/s12984-016-0176-z

**Published:** 2016-07-27

**Authors:** Michiel Punt, Sjoerd M. Bruijn, Kimberley S. van Schooten, Mirjam Pijnappels, Ingrid G. van de Port, Harriet Wittink, Jaap H. van Dieën

**Affiliations:** 1 Research group Lifestyle and Health, Utrecht University of Applied Sciences, Bolognalaan 101, Utrecht, 3584 JW The Netherlands; 2 Move Research Institute Amsterdam, Department of Human Movement Sciences, Vrije Universiteit Amsterdam, Amsterdam, The Netherlands; 3 Department of Orthopedics, first affiliated Hospital of Fujian Medical University, Fuzhou, Fujian People’s Republic of China; 4 Revant Rehabilitation Center Breda, Breda, The Netherlands

**Keywords:** Gait quality, Gait quantity, Accelerometry, Stability, Accidental falls, Fall risk

## Abstract

**Background:**

Falls in stroke survivors can lead to serious injuries and medical costs. Fall risk in older adults can be predicted based on gait characteristics measured in daily life. Given the different gait patterns that stroke survivors exhibit it is unclear whether a similar fall-prediction model could be used in this group. Therefore the main purpose of this study was to examine whether fall-prediction models that have been used in older adults can also be used in a population of stroke survivors, or if modifications are needed, either in the cut-off values of such models, or in the gait characteristics of interest.

**Methods:**

This study investigated gait characteristics by assessing accelerations of the lower back measured during seven consecutive days in 31 non fall-prone stroke survivors, 25 fall-prone stroke survivors, 20 neurologically intact fall-prone older adults and 30 non fall-prone older adults. We created a binary logistic regression model to assess the ability of predicting falls for each gait characteristic. We included health status and the interaction between health status (stroke survivors versus older adults) and gait characteristic in the model.

**Results:**

We found four significant interactions between gait characteristics and health status. Furthermore we found another four gait characteristics that had similar predictive capacity in both stroke survivors and older adults.

**Conclusion:**

The interactions between gait characteristics and health status indicate that gait characteristics are differently associated with fall history between stroke survivors and older adults. Thus specific models are needed to predict fall risk in stroke survivors.

## Background

Falls are a major problem in the growing older population. Falls can result in serious injuries leading to considerable medical costs [1]. In stroke survivors, fall rates are higher in comparison to healthy older adults [2, 3]. Falls may increase the fear of falling and may subsequently reduce physical activity [4], which can result in physical deconditioning and may further increase fall risk in the long term.

Objective fall risk assessment often involves assessment of balance control, for example with the Berg Balance Scale [5]. However, the relation between deficits in balance control and fall rates in stroke survivors is inconsistent [3, 5]. It has been suggested that this might be due to fact that most balance tests are static in nature [6], while most falls occur during dynamic tasks such as walking and transfers [3]. Interestingly, several characteristics of gait quality have been shown to differentiate fallers from non-fallers among older adults [7–9]. These characteristics can be measured in daily life by accelerometry, and reflect aspects such as stability, symmetry, smoothness and variability. van Schooten et al. [9]. demonstrated the added value of such gait characteristics to conventional clinical predictors of fall risk.

A similar approach may be useful for stroke survivors, and could add value to existing clinical tests. However, quantity and quality of gait are different in stroke survivors [10–12] than in healthy individuals, and it is therefore unclear whether a fall risk prediction model as used by van Schooten et al. [9] can be used in stroke survivors. For example, stroke survivors have a more asymmetrical [11] and unstable gait [10] compared to age matched controls. Furthermore stroke survivors are physically less active [12] and physical activity has been associated to falls as well [9]. Thus, even if the same gait quality characteristics predict falling in stroke survivors and in healthy controls, it may be that regression coefficients for these characteristics are markedly different in a prediction model for stroke survivors compared to models developed for healthy older adults.

To this date, exploring the potential of fall prediction models based on gait characteristics has been limited to older adults; however, gait in stroke survivors is remarkably different and fall incidences are a frequent problem in stroke survivors. To explore the potential of using daily life gait assessment to predict falls in stroke survivors, the main purpose of the current study was to examine whether fall-prediction models that have been used in healthy older adults [7–9] can also be used in a population of stroke survivors, or if modifications are needed, either in the regression coefficients of such models, or in the gait characteristics of interest.

## Methods

### Participants

We tested community-dwelling stroke survivors as well as healthy older adults. Stroke survivors were recruited via local physical therapy centers and through national peer group meetings. Stroke survivors were above the age of 18, at least 1 year post stroke and were living in the community independently. Stroke survivors were excluded from the study if they had a functional ambulation category of two or less. Data for the healthy older adults group were derived from a data set of a larger study on ‘fall risk assessment in older adults’ (FARAO) [8, 9]. We only included participants from who we were certain that they were free of any neurological damage, such as a history of stroke or Parkinson’s disease. Control participants for this study were recruited via general practitioners, pharmacies and residential care facilities in the area of Amsterdam, The Netherlands. Participants were excluded from the study if they had severe cognitive disorders, as indicated by a minimal mental state examination score of 24 or less [13]. All participants were able to walk independently for at least 20 m, if necessary with a walking aid. The research protocol was approved by the medical ethical committees of the University Medical Center Utrecht and the VU medical center Amsterdam, The Netherlands. All participants signed informed consent and treatment of the participants was according to Good Clinical Practice.

### Data collection

Fall status was determined using a self-reported questionnaire asking about falls in the last 12 months prior to determining gait characteristics. A fall was defined as; ‘any unanticipated event that results in participant coming to the ground, floor or lower level’ [14]. To estimate quantitative and qualitative gait characteristics, participants were asked to wear a tri-axial accelerometer (55 g), (McRoberts, Den Haag, The Netherlands) at the middle of the lower back using an elastic belt [15]. The accelerometer was aligned to coincide with the anterior-posterior (AP), medio-lateral (ML) and vertical (VT) body-axis. Participants were instructed to realign the accelerometer during the monitoring period if necessary. Data were sampled at 100 samples/s with a range ± 6 g and digitally stored on a mini SD card. Participants were instructed to wear the accelerometer for seven consecutive days, preferably during day and night, but were allowed to take it off when going to bed. The accelerometer was removed during showering and other water-related activities to prevent damage.

### Data analysis

Based on health status and fall history of at least one fall in the previous year, we classified participants into four groups: non fall-prone stroke survivors (NF-SS), fall-prone stroke survivors (F-SS) and a control (CON) group of older adults thus fall-prone older adults (F-CON) and non fall-prone older adults (NF-CON) and used these groups for further analyses.

Gait activity was identified from the weekly time series using a validated algorithm for gait detection and gait quantification [16]. The algorithm searches for gait activity based on the spectral content of the AP acceleration and discards periods of activity shorter than 8 s. Total monitoring time was defined as the time between the first and last gait episode of each participant over the 7 days. Prior to estimating gait characteristics, potential accelerometer misalignment was corrected according to the method described by Rispens et al. [15].

Gait quantity was expressed as the duration of gait activity per 24 h and the number of walking bouts per 24 h. We classified walking bouts of 24 s or shorter as short walking bouts. Short walking bouts are likely to be executed predominantly in at-home settings, which might affect gait characteristics (for instance, turning affects step length symmetry, and usually in-home walking coincides with more turning as compared to walking outdoors). All walking bouts were divided into 8-s epochs for further analysis. We calculated the number of 8 s epochs that were part of the short walking bouts and divided this by the total number of epochs. Thus we were able to determine whether the characteristics of gait quality were derived under similar circumstances between groups.

We estimated gait quality characteristics that have shown promise in differentiating between fallers and non-fallers among older adults [8]. Specifically, we calculated gait speed [17], gait symmetry determined by the harmonic ratio (HR) [18]. This HR measure divides the sum of the first ten even harmonics through the sum of the first ten odd harmonics. Symmetrical gait in the VT and AP direction will predominantly contain even harmonics which will result in a higher HR. The smoothness of gait was determined by dividing the ground frequency (first harmonic) of the time series by the first six harmonics of the time series, the index of harmonicity (IH) [19]. A complete smooth gait can be described by one sinusoidal function and no higher harmonics would be necessary to describe the signal. Subsequently this would result in a higher IH value. Several indicators of gait variability were determined. Firstly the amplitude of the dominant peak which represents the ‘strength’ of the dominant peak relative to the rest of the signal [20], and hence a high value represents a low variability. Secondly the width of peak of the power spectrum reflects the dispersion of the dominant peak [20] and hence a higher value represents a higher variability. Thirdly stride frequency variability, and fourthly local dynamic stability expressed as the local divergence exponent (LDE), which quantifies the exponential rate of divergence from initially nearby kinematics states as a function of stride time [8]. A higher LDE indicates a faster diverging acceleration signal and indicates a more unstable gait pattern. Except for gait speed and stride time all these characteristics were determined in three directions using algorithms previously described by Rispens et al. [8]. Estimation of gait quality characteristics was performed on each epoch of 8-s length, which was sufficient long for estimating spectral features [21]. For each characteristic, the median value over all gait epochs of a participant was used for statistical evaluation. We took the median value as the median is less sensitive in comparison to the mean for outliers in the estimated gait characteristic.

### Statistics

For each group, means and standard deviations are reported. Participant characteristics and monitoring duration were compared between groups using health status (stroke survivor or healthy older adult) and fall history as two categorical factors in an analysis of variance (ANOVA). When these analyses revealed differences between groups, the variable concerned was used as a covariate to control for its effects in subsequent analyses.

To facilitate objective comparison between independent variables we z transformed continues variables prior to performing the logistic regression. We developed a fall prediction model per gait characteristic using binary logistic regression. Fall history was used as dependent variable, while the gait characteristic and the categorical variable health status (stroke survivors versus healthy older adults, coded as 1 and 0 respectively) were the independent variables. The interaction between health status and the gait characteristic was also included in the model, but if the interaction did not reach a *p*-value of ≤ 0.05 it was removed from the model and a new model with health status and the gait characteristic only was created. The odds ratio (OR) is a number indicating the amount of increased fall risk per unit increase of the independent variable. If a significant OR is found for a specific gait characteristic only, this implies that it is associated with fall history and that this association does not depend on health status (controls, older adults and stroke survivors). If in addition a significant OR is found for health status, but no interaction, this implies that fall risk is dependent on health status, but that the change in risk with a unit change in the gait characteristic is independent of health status. If an interaction effect is found, this implies that the relation of the gait characteristic with fall history is different between health status groups, suggesting that a specific model is needed for stroke survivors. All statistical analyses were performed using SPSS software version 20.0, and a *p*-value of ≤ 0.05 was considered statistically significant.

## Results

A total of 106 participants volunteered for the study. Of the 56 participating stroke survivors 25 (45 %) had experienced at least one fall in the previous year. A total of 50 control older adults participated of whom 20 (40 %) had experienced at least one fall in the previous year. Participant characteristics and monitoring duration results for each group are presented in Table 1. Tables 2 and 3 provide an overview of which gait characteristics show promise in regard to predicting fall risk in stroke survivors and older adults. More precise, Table 2 provides an overview of quantitative and qualitative gait characteristic values between stroke survivors and older adults and is as well subdivided in fallers and non fallers. An overview of the corresponding Odds ratios (OR) between all four groups and *p*-values derived from the binary logistic regression models are presented in Table 3.

**Table 1:** Participant characteristics for the four groups

	NF-SS	F-SS	NF-CON	F-CON	Health status		Fall history		Interaction	
	Mean ± SD	Mean ± SD	Mean ± SD	Mean ± SD	F	*P*-value	F	*P*-value	F	*P*-value
Female/male	15/16	15/10	13/17	14/6						
Age (years)	64.1 ± 11.6	69.0 ± 9.2	71.9 ± 4.1	74.9 ± 8	14.98	**<0.001**	0.29	0.59	5.21	**0.024**
Height (m)	171.4 ± 8.8	172.3 ± 9.3	169.4 ± 9.2	170.4 ± 7.7	1.37	0.244	0.01	0.99	0.33	0.564
Weight (kg)	79.7 ± 14.7	82.9 ± 16.5	75.1 ± 10.5	75.3 ± 13.7	3.87	0.051	3.89	0.534	0.34	0.561
BMI (kg/m2)	27.0 ± 3.8	28.1 ± 6.5	25.8 ± 2.5	26.1 ± 3.7	3.52	0.063	0.71	0.400	0.21	0.645
Monitoring(days)	6.5 ± 0.5	6.5 ± 0.4	6.4 ± 0.7	6.5 ± 0.6	0.85	0.771	0.48	0.827	0.945	0.333

Main effects for health status and fall history and their interaction are presented. Significant *p*-values are printed in bold

*NF*-*SS* non-fallers, stroke survivors, *F*-*SS* faller, stroke survivor, *NF*-*CON* non-faller control group of older adults, *F*-*CON* faller control group of older adult

**Table 2:** Quantitative and qualitative gait characteristics for the four groups

	NF-SS	F-SS	NF-CON	F-CON
Quantitative measures	Mean ± SD	Mean ± SD	Mean ± SD	Mean ± SD
Gait activity (min/day)	35.9 ± 20.1	34.4 ± 24	47.5 ± 21.8	50 ± 19.8
WB/day	127 ± 64.5	125 ± 77.3	158 ± 54.4	152 ± 49.9
Short WB (%)	91.6 ± 4.8	92.4 ± 6.2	89.7 ± 6	90.2 ± 3.9
Short WB epochs(%)	27.8 ± 18.4	43.3 ± 28.5	25.5 ± 15.9	23.8 ± 10.1
Qualitative measures				
Gait speed (m/s)	0.74 ± 0.14	0.67 ± 0.17	0.93 ± 0.30	0.90 ± 0.21
Stride time (seconds)	1.26 ± 0.20	1.44 ± 0.40	1.14 ± 0.13	1.15 ± 0.08
**Harmonic Ratio VT**	1.31 ± 0.20	1.20 ± 0.20	1.92 ± 0.31	1.67 ± 0.32
Harmonic Ratio ML	1.33 ± 0.13	1.35 ± 0.22	1.45 ± 0.15	1.51 ± 0.26
**Harmonic Ratio AP**	1.17 ± 0.20	1.07 ± 0.20	1.71 ± 0.19	1.39 ± 0.24
Freq. Variability VT	0.15 ± 0.05	0.14 ± 0.03	0.14 ± 0.20	0.14 ± 0.02
Freq. Variability ML	0.18 ± 0.04	0.21 ± 0.05	0.16 ± 0.40	0.17 ± 0.04
Freq. Variability AP	0.17 ± 0.04	0.19 ± 0.05	0.14 ± 0.30	0.15 ± 0.04
**IH VT**	0.48 ± 0.18	0.39 ± 0.16	0.64 ± 0.12	0.56 ± 0.13
**IH ML**	0.38 ± 0.21	0.50 ± 0.25	0.34 ± 0.13	0.25 ± 0.15
IH AP	0.51 ± 0.15	0.52 ± 0.15	0.57 ± 0.11	0.52 ± 0.09
**Amplitude (psd) VT**	0.47 ± 0.12	0.40 ± 0.09	0.56 ± 0.09	0.58 ± 0.11
**Amplitude (psd) ML**	0.40 ± 0.15	0.50 ± 0.19	0.36 ± 0.08	0.36 ± 0.08
Amplitude (psd) AP	0.42 ± 0.11	0.46 ± 0.17	0.42 ± 0.09	0.41 ± 0.08
**Width (psd) VT**	0.98 ± 0.01	0.99 ± 0.02	0.95 ± 0.01	0.94 ± 0.01
Width (psd) ML	0.95 ± 0.02	0.95 ± 0.03	0.95 ± 0.01	0.95 ± 0.01
Width (psd) AP	0.95 ± 0.02	0.95 ± 0.01	0.94 ± 0.01	0.93 ± 0.01
LDE/stride VT	0.98 ± 0.19	1.02 ± 0.19	0.92 ± 0.15	0.94 ± 0.16
**LDE/stride ML**	0.89 ± 0.18	0.90 ± 0.20	0.71 ± 0.08	0.78 ± 0.11
LDE/stride AP	0.87 ± 0.19	0.90 ± 0.21	0.72 ± 0.10	0.78 ± 0.11

Table 2, Quantitative and qualitative gait characteristics for all four groups expressed as means and standard deviations. *Abbreviations*: WB is walking bouts, Freq. Variability is the stride frequency variability, IH is the Index of Harmonicity, Amplitude (psd) is the amplitude of the dominant peak in the power spectral density, Width (psd) is the width of the dominant peak in the power spectral density, LDE/stride is the local divergence exponent per stride. VT is the vertical direction, ML is the medio-lateral direction and AP is the anterior-posterior direction. Significant associations derived from logic regression (Table 3) are printed in bold

**Table 3:** Binary logistic regression

	Gait characteristic	Health status	Interaction
Quantitative measures	OR (*p*)	OR (*p*)	OR (*p*)
Gait activity (min/day)	1.01 (.94)	0.82 (.63)	
WB/day	0.94 (.74)	0.85 (.69)	
Short WB (%)	1.17 (.43)	0.88 (.76)	
Short WB epochs(%)	1.48 (.06)	0.99 (.99)	
Qualitative measures			
Gait speed (m/s)	0.64 (.06)	1.17 (.71)	
Stride time (seconds)	1.71 (.07)	1.19 (.68)	
Harmonic Ratio VT	**0.54 (.02)**	1.61 (.33)	
Harmonic Ratio ML	1.23 (.31)	0.71 (.41)	
Harmonic Ratio AP	**0.34 (<.01)**	2.72 (.06)	
Freq. Variability VT	0.81 (.34)	0.81 (.59)	
Freq. Variability ML	1.45 (.08)	1.01 (.94)	
Freq. Variability AP	1.41 (.12)	1.08 (.86)	
IH VT	**0.46 (<.01)**	1.81 (.23)	
IH ML	**1.64 (.05)**	0.65 (.37)	**0.22(<.01)**
IH AP	0.84 (.40)	0.87 (.73)	
Amplitude (psd) VT	**0.44 (.02)**	1.21 (.70)	**2.76 (.04)**
Amplitude (psd) ML	**1.54 (.04)**	1.05 (.91)	
Amplitude (psd) AP	1.04 (.83)	0.90 (.81)	
Width (psd) VT	1.11 (.63)	**0.09 (.04)**	**0.01 (.02)**
Width (psd) ML	0.98 (.94)	0.82 (.62)	
Width (psd) AP	0.87 (.51)	0.81 (.61)	
LDE/stride VT	1.31 (.21)	1.02 (.97)	
LDE/stride ML	1.16 (.54)	2.23 (.13)	**4.86 (.03)**
LDE/stride AP	1.54 (.06)	1.31 (.55)	

Quantitative and qualitative gait characteristics association with a history of falls. Health status includes stroke survivors versus older adults. Data was z-transformed prior to logistic regression. Significant associations are printed in bold

Two-way ANOVAs for participant characteristics revealed no differences in monitoring duration and anthropometrics between groups, but showed a significant interaction effect on age, with the NF-SS group being significantly younger than the other three groups. Further results were corrected for this difference by using age as covariate.

No associations with a history of falls were found for gait quantity characteristics (see Table 3). Four gait quality characteristics were found to be associated with a history of falls independent of health status (see Table 3). A reduced gait symmetry (HR) in the VT and AP direction and decreased gait smoothness (IH) in the VT direction were associated with a history of falls in both groups. Moreover an increase in the dominant amplitude of the power spectrum in the ML direction was associated with an increased fall risk in both groups. Increased stride time and reduced gait speed showed a trend of increased fall risk in both groups, respectively (*p* = .06 and *p* = .07).

In addition for four gait quality characteristics a significant interaction term between health status and gait characteristic, was predictive for fall history (see Table 3). For gait smoothness (IH) in the ML direction, this indicated that a higher IH increased fall risk, but less so in stroke patients, although stroke increased the fall risk. Moreover an larger width of the dominant peak of the power spectrum in VT direction higher fall risk in stroke survivors but lower in older adults. A higher amplitude of the dominant peak in VT was associated with a lower fall risk in stroke survivors but not in older adults, with stroke increasing fall risk as well. Finally, an increase in the local divergence exponent (decrease in local dynamic stability) in the ML direction, increased fall risk in stroke survivors, but not in healthy older adults.

## Discussion

The main purpose of the study was to test whether fall-prediction models that have been used in healthy older adults can also be used in a population of stroke survivors, or if modifications are needed, either in the regression coefficients, or in the gait characteristics of interest.

Previous studies assessing gait in older adults have shown that gait speed [22], gait variability [23], local dynamic stability [8, 9, 24] and symmetry [25] provide valuable information concerning fall risk in older adults. Our results are partly in line with these findings as we found several similar gait characteristics that were able to predict falls in both groups. However the limited number of participants in our study reduced statistical power in comparison to previous work [8, 9], which could explain that not all findings are reproduced in this study. For instance gait speed and stride time showed only a trend of predictive ability in both groups, respectively (*p* = .06 and *p* = .07).

Interestingly, we found four interactions indicating a different relation between gait quality and fall history in stroke survivors compared to the group of healthy older adults. In addition gait symmetry in the AP direction was predictive for falls in both groups, but health status showed a trend (*p* = .06) indicating a different cut off value in the regression. Thus, since we found gait characteristics that were predictive for falls in both groups but we also found gait characteristics with a interaction or different cut off value the overall results indicate that predicting falls in stroke survivors based on daily-life gait characteristics is possible but requires a stroke specific fall-prediction model.

Surprising results were found for the index of harmonicity, which is calculated by dividing the power of the fundamental frequency by the power of the first six harmonics. This measure is thought to reflect the smoothness of gait. Interestingly, based on mean and standard deviations between groups the F-SS group had the highest index of harmonicity in the ML direction but the lowest in the AP and VT directions. This different relation between ML and AP, VT direction for the index of harmonicity can possibly be explained by a more pronounced and rapid shift of the center of mass between both legs in this group, to reduce the time standing on the paretic leg. Such fast movement results in a high peak in the acceleration signal at the stride frequency. Therefore the index of harmonicity in the ML direction, measured at the pelvis might reflect more a rigid gait pattern and loss of control in the paretic leg rather than smoothness of gait. Considering the present differences between groups, it is a gait characteristic of interest in the stroke population. Moreover, this opposite relation between ML and AP, VT direction for the index of harmonicity was confirmed by our findings on the amplitude of the dominant peak in the power spectrum. The amplitude of the dominant peak was highest for the F-SS group in the ML direction but lowest in the VT direction, which is in line with the findings by Weiss et al. [7].

Although none of the gait quantity characteristics were associated with a history of falls, gait activity and the number of walking bouts seems to be reduced in stroke survivors, see also Table 2. Since most falls occur during gait [26], this reduced gait activity could be a fall risk avoidance strategy. However reduced gait activity may cause further deconditioning and subsequently increased fall risk in the long-term.

### Study limitations

To divide our groups of interest into fallers and non-fallers we have used self-reported retrospective fall incidents. Retrospective fall reports can be influenced by recall bias and their relation with gait quality may slightly differ from prospective fall reports [9]. These differences may have influenced our classification. Second, the identification of gait epochs for estimating gait characteristics was accomplished by a gait detection algorithm [16]. Although validity and reliability was good for slow and fast walking, it still remains unknown to what extent the algorithm identified other forms of cyclic movements such as biking. Misclassifications of gait activity or for instance wearing the accelerometer away from the midline of the lower back will result in deviating estimations of gait characteristics for those epochs. Yet the error in our final estimating gait characteristics is limited by taking the median value over all epochs rather than the mean. Third, the results showed differences in percentages of short walking bouts between groups. This suggests that the median value for the qualitative gait characteristics were estimated based on slightly different environmental circumstances. This is an important finding, because for example gait symmetry may be affected by bends and shorter walking bouts are probably performed in a more complex setting, which contains more bends. To examine whether this finding influenced our results we compared gait characteristics between groups including only walking bouts lasting 16 s or more. Mean values were somewhat different but no consistent changes were found and the main findings would have been the same as presented here. In addition we reanalyzed our statistical models taking weight and BMI as covariates, since both variables were nearly significant different between groups. OR were slightly different yet the same interactions were still present.

## Conclusion

In conclusion, due to the present interactions found, several gait characteristics are differently associated with a history of falls in stroke survivors as in older adults. This suggests that specific models are needed to predict fall risk in stroke survivors.

## Abbreviations

AP, anterior-posterior; BMI, body mass index; F-CON, fall prone controls; F-SS, fall-prone stroke survivors; HR, harmonic ratio; IH, index of harmonicity; LDE, local divergence exponent; ML, medio-lateral; NF-CON, non fall prone controls; NF-SS, non fall-prone stroke survivors; OR, odds ratio; VT, vertical
